# A 10-year review of pediatric inguinal hernia management at a tertiary center

**DOI:** 10.3389/fped.2026.1802302

**Published:** 2026-04-20

**Authors:** Bingran Yu, Shuan Liu, Zai Song

**Affiliations:** 1 Department of Pediatric Surgery, Children's Hospital of Fudan University, and Shanghai Key Laboratory of Birth Defects, Shanghai, China; 2 Xiamen Key Laboratory of Pediatric General Surgery Diseases, Children's Hospital of Fudan University (Xiamen Branch), Xiamen Children's Hospital, Xiamen, China

**Keywords:** incarceration, laparoscopic inguinal hernia repair, open inguinal hernia repair, pediatric inguinal hernia, recurrence

## Abstract

**Aims:**

To analyze the epidemiology, clinical characteristics, surgical outcomes, and risk factors for incarceration and recurrence in a large cohort of children undergoing inguinal hernia repair, and to evaluate differences across sex, laterality, and surgical approaches.

**Methods:**

We retrospectively reviewed the children who underwent inguinal hernia repair at a tertiary pediatric center over ten years. Demographic characteristics, perioperative outcomes, and postoperative complications were analyzed. Subgroup comparisons were performed by sex, laterality, and surgical approach. Multivariate logistic regression models were used to identify independent predictors of incarceration and recurrence.

**Results:**

Of 9590 children, 72.2% were male, and the median age was 2 years and 10 months. Laparoscopic surgery was performed in 93.1% of cases. Incarceration occurred in 4.2% of children and recurrence in 1.4%. Females, children ≤1 year, and unilateral hernias were independently associated with higher risk of incarceration. Male sex and age >1 year predicted recurrence, while laparoscopic technique served as a protective factor. Laparoscopy identified synchronous contralateral hernias in 39.2% of children initially diagnosed with unilateral hernia, compared with only 0.9% detected during open repair. Laparoscopic approach was also associated with shorter operative time, fewer complications, and faster recovery.

**Conclusions:**

This large cohort study highlights the epidemiology and surgical outcomes of pediatric inguinal hernia. Age, sex, and hernia laterality were associated with clinical presentation and complication risk. Laparoscopic surgery showed favorable perioperative outcomes and facilitated detection of contralateral hernias. Because surgical approach selection was not randomized, comparisons between techniques should be interpreted cautiously. These findings emphasize the importance of individualized risk stratification and surgical decision-making.

## Introduction

Inguinal hernia is one of the most common pediatric surgical conditions, which affects 0.8–4.4% of all children (1). It typically results from the failure of processus vaginalis closure and presents as a reducible mass in the inguinal region or scrotum. Although the condition is benign in most cases, inguinal hernia may progress to incarceration or strangulation, warranting timely surgical intervention (2).

The standard treatment for inguinal hernia is surgical repair, and various techniques have been adopted over time, including the open and laparoscopic approaches (3). Traditionally, the open approach has been widely used and is considered a reliable technique with low recurrence rates. Over the past two decades, the laparoscopic approach has gained increasing popularity because of its minimally invasive nature, improved cosmetic outcomes, and the ability to inspect the contralateral internal ring (4).

This retrospective study was conducted to provide a comprehensive evaluation of pediatric inguinal hernia management in a large single-center cohort. Specifically, we aimed to describe the epidemiological and clinical characteristics of pediatric inguinal hernia, compare perioperative outcomes between open and laparoscopic approaches in real-world clinical practice, and identify independent risk factors associated with incarcerated and recurrent hernia.

## Materials and methods

### Patient cohorts

This retrospective study was conducted in accordance with the Declaration of Helsinki and approved by the Institutional Review Board of Children's Hospital of Fudan University. The medical records of children receiving inguinal hernia repair at the Department of Pediatric Surgery of Children's Hospital of Fudan University from 2,016 to the present were reviewed. Children were excluded if they underwent additional surgical procedures at the time of inguinal hernia surgery.

### Treatment protocol

The choice between open and laparoscopic approaches was guided by clinical presentation and predefined operative considerations, including the severity of incarceration, reducibility of hernia contents, and anticipated operative complexity.

In the open approach, a transverse incision was made in the inguinal region. The tissue, muscle and fascia were separated to identify the hernia sac. The sac was opened to confirm whether there were herniated contents. After transection of the sac's posterior wall, the proximal sac was mobilized to the level of the extraperitoneal fat and high ligated at the neck with two passes of 4-0 silk.

All laparoscopic procedures in this study were performed using single-site laparoscopic percutaneous extraperitoneal closure (5, 6). A transumbilical incision was made to access the abdominal cavity, followed by insertion of a 5-mm trocar, establishment of pneumoperitoneum and insertion of a laparoscope. A hernia needle loaded with double sutures was inserted into the extraperitoneal space. After passing a half circle around the neck of the hernia sac, the peritoneum was punctured and the sutures were left in the abdominal cavity. The needle was then reinserted from the same puncture site and passed laterally around the sac to retrieve the sutures, completing a peritoneal loop. After confirming proper positioning, the sutures were tied extracorporeally to achieve high ligation.

During laparoscopic approach, the contralateral processus vaginalis was routinely evaluated. A contralateral processus vaginalis was considered patent when an obvious open internal ring was visualized, with or without peritoneal protrusion or passage of fluid/air bubbles. In such cases, simultaneous contralateral repair was performed using the same technique.

All procedures were performed by experienced pediatric surgeons or under their direct supervision. Surgeons performing laparoscopic approach had completed standardized training in pediatric minimally invasive surgery and were proficient in the described technique.

### Statistics analysis

All statistical analyses were performed using SPSS version 26.0. Continuous variables were presented as median with range, and categorical variables were expressed as frequencies and percentages. The Chi-square test or Fisher's exact test was used for categorical variables, and the independent-samples t-test or Mann–Whitney U test for continuous variables. The Kruskal–Wallis H test was used for comparing more than two groups. Multivariate logistic regression analyses were performed to identify independent risk factors for incarcerated and recurrent inguinal hernia. Variables with *P* < 0.05 in univariate analyses were included in the multivariate models. Hazard ratios (HR) with 95% confidence intervals (CIs) were calculated. A two-sided *P* < 0.05 was considered statistically significant.

## Results

### Patient characteristics

A total of 9,590 children with inguinal hernia were included in the analysis, consisting of 6,928 males (72.2%) and 2,662 females (27.8%) (Supplementary Table S1). The median age at the time of surgery was 2 years and 10 months (range: 16 days−17 years). Among all children, 137 (1.4%) were diagnosed as recurrent hernia. Regarding hernia laterality, 21.6% were left-sided, 33.4% right-sided, and 45.0% bilateral.

In terms of surgical approach, 649 cases (6.8%) underwent the open approach, while 8,931 (93.1%) received the laparoscopic approach. Additionally, 10 children who were initially scheduled for the laparoscopic approach required intraoperative conversion to the open surgery. The main indications included difficulty in reduction of incarcerated contents (7 cases), unclear anatomy of the hernia sac (2 cases), and technical challenges during extraperitoneal suturing (1 case). The median operative time was 29 min (range: 10–188 min). The surgical complications occurred in 11 patients (0.1%), and the median length of postoperative hospital stay was 1 day (range: 1–28 days).

Among children initially diagnosed with unilateral hernia, 3,508 cases (39.2%) were found intraoperatively to have bilateral defects.

### Comparison of surgical approaches

Baseline characteristics and perioperative outcomes between the open and laparoscopic approaches are summarized in Table 1. Patients who underwent the open approach were younger than those treated with the laparoscopic approach (*P* < 0.001). The open group also exhibited a significantly higher proportion of recurrent hernias (3.7% vs. 1.3%, *P* < 0.001), and incarcerated hernias (33.0% vs. 2.1%, *P* = 0.001) than the laparoscopic group.

**Table 1:** Comparison between open and laparoscopic approaches for inguinal hernia.

Variables	Open (*n* = 649)	Laparoscopic (*n* = 8,941)	*P* value
Sex			0.024
Male	444 (68.4)	6,484 (72.5)	
Female	205 (31.6)	2,457 (27.5)	
Age^a^	1y11 m (16d−15y)	2y11 m (17d−17y)	< 0.001
Type of hernia			< 0.001
Recurrent hernia	24 (3.7)	113 (1.3)	
non-Recurrent hernia	625 (96.3)	8,828 (98.7)	
Metachronous contralateral hernia			< 0.001
Yes	30 (4.6)	69 (0.8)	
No	619 (95.4)	8,872 (99.2)	
Incarcerated hernia			0.001
Yes	214 (33.0)	184 (2.1)	
No	435 (67.0)	8,757 (97.9)	
History of abdominal surgery			< 0.001
Presence	44 (6.8)	134 (1.5)	
Absence	605 (93.2)	8,807 (98.5)	
Side of hernia			< 0.001
Unilateral	591 (91.1)	4,687 (52.4)	
Left	268 (41.3)	1,803 (20.2)	
Right	323 (49.8)	2,884 (32.2)	
Bilateral	58 (8.9)	4,254 (47.6)	
Operative time (min)	35 (10–180)	28 (10–188)	<0.001
Synchronous contralateral hernia			<0.001
Presence	4 (0.6)	3,504 (39.2)	
Absence	645 (99.4)	5,437 (60.8)	
Surgical complications			<0.001
Presence	7 (1.1)	4 (0)	
Absence	642 (98.9)	8,937 (100)	
Length of postoperative hospital stay (days)	1 (1–28)	1 (1–20)	<0.001

a d, days; m, months; y, years.

The operative time was slightly longer in the open group (*P* < 0.001). In contrast, the laparoscopic approach enabled intraoperative identification of synchronous contralateral hernias in 39.2% of patients initially diagnosed with unilateral hernia, compared with 0.9% detected during the open approach (*P* < 0.001). Postoperative complications were rare in both groups, and the median length of postoperative hospital stay was similar, although the range was wider in the open group.

### Sex-based comparison of inguinal hernia

Baseline characteristics and perioperative outcomes were compared between 6,928 males and 2,662 females (Table 2). Male patients were significantly younger at the time of surgery than females (*P* < 0.001). Recurrent hernia (1.7% vs. 0.8%, *P* = 0.001) and metachronous contralateral hernia (1.2% vs. 0.6%, *P* = 0.018) occurred more frequently in males, while incarcerated hernia was more common in female patients (5.0% vs. 3.8%, *P* = 0.010).

**Table 2:** Comparison between male and female patients with inguinal hernia.

Variables	Male (*n* = 6,928)	Female (*n* = 2,662)	*P* value
Age^a^	2y2 m (17d−16y)	4y8 m (16d−17y)	<0.001
Type of hernia			0.001
Recurrent hernia	116 (1.7)	21 (0.8)	
non-Recurrent hernia	6,812 (98.3)	2,641 (99.2)	
Metachronous contralateral hernia			0.018
Yes	82 (1.2)	17 (0.6)	
No	6,846 (98.8)	2,645 (99.4)	
Incarcerated hernia			0.010
Yes	265 (3.8)	133 (5.0)	
No	6,663 (96.2)	2,529 (95.0)	
History of abdominal surgery			<0.001
Presence	151 (2.2)	27 (1.0)	
Absence	6,777 (97.8)	2,635 (99.0)	
Side of hernia			<0.001
Unilateral	3,913 (56.5)	1,365 (51.3)	
Left	1,443 (20.8)	628 (23.6)	
Right	2,470 (35.7)	737 (27.7)	
Bilateral	3,015 (43.5)	1,297 (48.7)	
Type of surgery			0.037
Open	444 (6.4)	205 (7.7)	
Laparoscopic	6,475 (93.5)	2,456 (92.3)	
Laparoscopic to Open^b^	9 (0.1)	1 (0.0)	
Operative time (min)	30 (10–180)	26 (10–188)	<0.001
Synchronous contralateral hernia			<0.001
Presence	2,495 (36.0)	1,013 (38.1)	
Absence	4,433 (64.0)	1,649 (61.9)	
Surgical complications			0.040
Presence	11 (0.2)	0 (0)	
Absence	6,917 (99.8)	2,662 (100)	
Length of postoperative hospital stay (days)	1 (1–22)	1 (1–28)	0.878

a d, days; m, months; y, years.

b Conversion from the laparoscopic surgery to the open surgery.

In terms of hernia laterality, bilateral hernias were more common in females (48.7% vs. 43.5%, *P* < 0.001), while right-sided hernias predominated in males (35.7% vs. 27.7%). The conversion from the laparoscopic surgery to the open surgery occurred in 1 female case, compared to 9 male cases. Operative time was slightly longer in males than in females (30 vs. 26 minutes, *P* < 0.001). Synchronous contralateral hernias were identified in a similar proportion of male and female patients during laparoscopic exploration. Surgical complications were rare and occurred only in males. No significant difference in length of postoperative hospital stay was observed between the two groups (*P* = 0.878).

### Laterality-based comparison

Clinical characteristics and surgical outcomes were further analyzed according to hernia laterality (Table 3). Male patients accounted for the majority in all groups, with the highest proportion in the right-sided hernia group. Patients with left-sided hernia were older compared with those with right-sided and bilateral hernia (*P* < 0.001). Recurrent hernias occurred more frequently in unilateral cases, whereas bilateral hernias demonstrated the lowest proportions of recurrence (0.4%) and contralateral metachronous hernia (0.1%). Incarcerated hernia occurred most frequently in left-sided cases, followed by right-sided and bilateral (6.0% vs. 4.6% vs. 2.9%, *P* < 0.001).

**Table 3:** Comparison among patients with left-sided, right-sided, and bilateral hernia.

Variables	Left (*n* = 2,071)	Right (*n* = 3,207)	Bilateral (*n* = 4,312)	*P* value
Sex				<0.001
Male	1,443 (69.7)	2,470 (77.0)	3,015 (69.9)	
Female	628 (30.3)	737 (23.0)	1,297 (30.1)	
Age^a^	3y5 m (21d−15y)	2y9 m (16d−16y)	2y9 m (17d−17y)	<0.001
Type of hernia				<0.001
Recurrent hernia	52 (2.5)	68 (2.1)	17 (0.4)	
non-Recurrent hernia	2,019 (97.5)	3,139 (97.9)	4,295 (99.6)	
Metachronous contralateral hernia				<0.001
Yes	50 (2.4)	45 (1.4)	4 (0.1)	
No	2,021 (97.6)	3,162 (98.6)	4,308 (99.9)	
Incarcerated hernia				<0.001
Yes	125 (6.0)	146 (4.6)	127 (2.9)	
No	1,946 (94.0)	3,061 (95.4)	4,185 (97.1)	
History of abdominal surgery				<0.001
Presence	63 (3.0)	85 (2.7)	30 (0.7)	
Absence	2,008 (97.0)	3,122 (97.3)	4,282 (99.3)	
Type of surgery				<0.001
Open	268 (12.9)	323 (10.1)	58 (1.3)	
Laparoscopic	1,801 (87.0)	2,880 (89.8)	4,250 (98.6)	
Laparoscopic to Open^b^	2 (0.1)	4 (0.1)	4 (0.1)	
Operative time (minutes)	26 (10–180)	26 (10–161)	32 (10–188)	<0.001
Surgical complications				0.815
Presence	2 (0.1)	3 (0.1)	6 (0.1)	
Absence	2,069 (99.9)	3,204 (99.9)	4,306 (99.9)	
Length of postoperative hospital stay (days)	1 (1–22)	1 (1–28)	1 (1–28)	<0.001

a d, days; m, months; y, years.

b Conversion from the laparoscopic surgery to the open surgery.

The surgical approach varied substantially by hernia laterality, with the laparoscopic approach predominating in all groups. The median operative time was longest in the bilateral hernia group. Surgical complications were rare and did not differ significantly among groups (*P* = 0.815). Length of postoperative hospital stay were generally short in all groups.

### Comparison and risk factor analysis of incarcerated inguinal hernia

Among the cohort, 398 patients (4.2%) presented with incarcerated inguinal hernia. Clinical characteristics of incarcerated hernia and comparison between surgical approaches are summarized in Table 4. Patients undergoing the open approach were generally younger and more likely to present with complex hernia contents, including intestinal contents (33.6% vs. 12.5%, *P* < 0.001) and ovarian or fallopian tube involvement (34.6% vs. 14.7%, *P* < 0.001). Operative time was significantly longer in the open group (33 min vs. 48 min, *P* < 0.001), while the laparoscopic approach allowed identification of synchronous contralateral hernias in 48.4% of patients.

**Table 4:** Comparison between open and laparoscopic approaches for incarcerated hernia.

Variables	Incarcerated hernia (*n* = 398)	Open (*n* = 214)	Laparoscopic (*n* = 184)	*P* value
Sex				0.025
Male	265 (66.6)	132 (61.7)	133 (72.3)	
Female	133 (33.4)	82 (38.3)	51 (27.7)	
Age^a^	10m16d (16d−12y)	8m23d (16d−9y)	1y1 m (21d−12y)	<0.001
Metachronous contralateral hernia				0.126
Yes	2 (0.5)	0 (0)	2 (1.1)	
No	396 (99.5)	214 (100)	182 (98.9)	
History of abdominal surgery				0.653
Presence	3 (0.8)	2 (0.9)	1 (0.5)	
Absence	395 (99.2)	212 (99.1)	183 (99.5)	
Side of hernia				<0.001
Unilateral	271 (68.1)	193 (90.2)	78 (42.4)	
Left	125 (31.4)	94 (43.9)	31 (16.8)	
Right	146 (36.7)	99 (46.3)	47 (25.5)	
Bilateral	127 (31.9)	21 (9.8)	106 (57.6)	
Operative time (minutes)	40.5 (10–180)	48 (10–180)	33 (10–143)	<0.001
Synchronous contralateral hernia				<0.001
Presence	91 (22.9)	2 (0.9)	89 (48.4)	
Absence	307 (77.1)	212 (99.1)	95 (51.6)	
Hernia contents				<0.001
Intestine	95 (23.9)	72 (33.6)	23 (12.5)	
Ovary or Fallopian tube	101 (25.4)	74 (34.6)	27 (14.7)	
Omentum	12 (3.0)	7 (3.3)	5 (2.7)	
Ileocecal region or Appendix	50 (12.6)	33 (15.4)	17 (9.2)	
Descending or sigmoid colon	7 (1.8)	5 (2.3)	2 (1.1)	
No content	133 (33.4)	23 (10.7)	110 (59.8)	
Surgical complications				0.189
Presence	2 (0.5)	2 (0.9)	0 (0)	
Absence	396 (99.5)	212 (99.1)	184 (100)	
Hospital length after surgery (days)	2 (1–28)	3 (1–28)	1 (1–14)	<0.001

a d, days; m, months; y, years.

Multivariate analysis identified several independent predictors of incarceration (Table 5). Female (HR = 1.926, 95% CI: 1.525–2.432, *P* < 0.001), age ≤1 year (HR = 0.071, 95% CI: 0.057–0.089, *P* < 0.001), and unilateral hernia (HR = 0.472, 95% CI: 0.376–0.591, *P* < 0.001) were independent predictors of incarceration.

**Table 5:** Univariate and Multivariate Analyses of Variables Associated with Incarcerated Hernia

Variables	Univariate	Multivariate	
	*P* value	*P* value	HR (95%CI)
Sex (Female/Male)	0.010	<0.001	1.926 (1.525–2.432)
Age (>1y/≤1y)	<0.001	<0.001	0.071 (0.057–0.089)
Type of hernia (non-Recurrent/Recurrent)	0.014	NS	
Metachronous contralateral hernia (No/Yes)	0.285		
History of abdominal surgery (Absence/Presence)	0.096		
Side of hernia (Bilateral/Unilateral)	<0.001	<0.001	0.472 (0.376–0.591)

HR Hazard ratio; CI Confidence Interval; NS not significant.

### Clinical features and risk factors of recurrent inguinal hernia

A total of 137 patients (1.4%) developed recurrent inguinal hernia (Supplementary Table 2). The majority were male (84.7%), and most (92.7%) experienced a single recurrence, with a median recurrence interval of 266 days (range: 2–3,391 days). Most recurrent hernias initially presented with unilateral hernia, and the majority remained unilateral after recurrence. Regarding surgical approach, 59.1% of patients were treated with the laparoscopic approach initially, whereas 82.5% received the laparoscopic surgery during reoperation. Surgical complications were rare, occurring in only one case (0.7%).

Multivariate analysis identified several independent predictors of recurrence (Table 6). Male sex (HR = 0.471, 95% CI: 0.293–0.756, *P* = 0.002) and age >1 year (HR = 9.826, 95% CI: 3.098–31.164, *P* < 0.001) were significantly associated with an increased risk of recurrence. Bilateral hernias were associated with a reduced risk of recurrence compared to unilateral hernias (HR = 0.258, 95% CI: 0.150–0.443, *P* < 0.001). Importantly, patients who underwent the laparoscopic surgery initially had a significantly lower risk of recurrence compared to those treated with the open surgery (HR = 0.121, 95% CI: 0.084–0.175, *P* < 0.001).

**Table 6:** Univariate and multivariate analyses of variables associated with recurrent hernia

Variables	Univariate	Multivariate	
	*P* value	*P* value	HR (95%CI)
Sex (Female/Male)	0.010	0.002	0.471 (0.293–0.756)
Age (>1 year/≤1 year)	<0.001	<0.001	9.826 (3.098–31.164)
Side of hernia before recurrence (Bilateral/Unilateral)	<0.001	<0.001	0.258 (0.150–0443)
Type of surgery before recurrence (Laparoscopic/Open)	<0.001	<0.001	0.121 (0.084–0.175)

HR, Hazard Ratio; CI, Confidence Interval.

## Discussion

In this comprehensive retrospective analysis of 9,590 children who underwent inguinal hernia repair over 10-year period at a tertiary center, we delineated the demographic characteristics, surgical patterns, and clinical outcomes associated with inguinal hernia in children. The exceptionally large cohort allowed us to assess subtle but meaningful differences across sex, laterality, and surgical approaches, and to identify independent predictors for incarceration and recurrence with high statistical robustness. These findings contribute substantially to the current understanding of pediatric inguinal hernia and offer implications for optimizing clinical decision-making.

One of the most striking observations in our study is the overwhelming predominance of the laparoscopic surgery, which accounted for 93.1% of all cases and represents one of the largest cohorts of laparoscopic participants reported to date (5, 7–9) This reflects a substantial shift in pediatric surgical practice over the past decade, during which the laparoscopic techniques have gradually replaced the open surgery as the preferred approach in many centers (6, 10, 11). Several previous studies have also demonstrated a growing preference for the laparoscopic surgery due to advantages such as improved visualization, reduced tissue trauma, and favorable cosmetic outcomes (8, 12–14).

An important advantage of laparoscopy demonstrated in this study is the ability to detect synchronous contralateral hernias (15). Among children preoperatively diagnosed with unilateral hernia, 39.2% were found to have bilateral defects intraoperatively during the laparoscopic approach, compared with 0.9% detected during the open surgery. From a clinical perspective, the capability of laparoscopy to reliably identify synchronous contralateral hernias is meaningful, as it allows for simultaneous high ligation and may prevent the development of metachronous contralateral hernia, which would otherwise necessitate a second operation and exposure to anesthesia (16, 17).

Our results also revealed notable sex-based differences. Although the majority of cases occurred in males, consistent with the well-established epidemiology of inguinal hernia, females demonstrated a significantly higher risk of incarceration. This was further supported by our multivariable analysis, which showed that the incidence of incarceration was 1.926 times higher in females than in males. Similar findings have been reported by Wang et al. (6), who also observed a greater likelihood of incarcerated hernia among female patients. Therefore, early surgical intervention should be strongly considered for female patients with inguinal hernia, even in the absence of overt symptoms. In addition, female patients were more likely to present with bilateral hernia. Accordingly, the laparoscopic surgery may be particularly appropriate for female patients and is also associated with a shorter operative time in females compared with males (6).

Laterality also emerged as an important determinant of clinical presentation. Right-sided hernia had the highest proportion of male patients, likely owing to the later and longer course of testicular descent, which predisposes the right processus vaginalis to delayed closure (18, 19). Children with left-sided hernias were significantly older and exhibited the highest incidence of incarceration. The explanation of this finding might be that left-sided hernias tend to be diagnosed later than right-sided hernias because they are less common, which may delay early elective repair and increase the risk of emergency presentations (20). These laterality-based differences emphasize that the anatomical side of the hernia may influence both risk stratification and surgical planning.

Incarcerated inguinal hernia occurred in 4.2% of our cohort, consistent with pediatric rates previously reported in a nation-wide longitudinal population-based study (21). In our analysis, a greater number of children with incarcerated hernia underwent the open surgery. This finding likely reflects differences in case selection in which patients selected for the open surgery tended to present with more complex or urgent conditions, characterized by a higher incidence of herniated contents such as intestine, adnexal structures, or the ileocecal region and appendix, rather than intrinsic differences between surgical techniques. During the open approach, direct exposure of the inguinal canal allows safer and more controlled handling of compromised bowel or adnexal tissues, making it advantageous in cases with significant incarcerated contents (22). Conversely, for children without significant hernia contents, laparoscopic surgery may be more appropriate and is associated with shorter operative times and faster postoperative recovery. However, this interpretation should be made cautiously because surgical allocation in this cohort was not randomized.

Multivariate analysis identified several factors independently associated with incarcerated hernia. In addition to female sex, age ≤1 year and unilateral hernia were significant predictors. Consistent with our findings, Wang et al.(6) also reported a higher likelihood of incarceration in children under 1 year of age. Younger patients are known to be more vulnerable to incarceration because the processus vaginalis remains widely patent during infancy. Furthermore, infants may be less able to communicate symptoms, which can delay recognition of early hernia signs (6). Therefore, greater attention should be directed toward female patients under 1 year of age to reduce the risk of severe incarcerated hernias. In addition, our findings also identified unilateral inguinal hernia as an independent risk factor for incarceration. This observation is consistent with clinical experience, as unilateral hernias often progress insidiously and may be overlooked at an early stage, particularly when symptoms are mild or intermittent. In contrast, bilateral hernias are more likely to be detected promptly due to more noticeable groin asymmetry or recurrent swelling, leading to earlier elective intervention. However, this finding requires further investigation in future studies.

The recurrence rate observed in this study was relatively low and consistent with previously reported pediatric series, in which recurrence rates typically range between 0.7% and 1.5% (7, 9, 23). In the study of Miyake et al. (24), recurrence occurred in 0.48% of cases, and all affected patients were male. Chen et al. (25) further reported that age > 3 years increased the risk of postoperative recurrence following laparoscopic high ligation of the hernia sac in children. Similarly, our analysis demonstrated male sex and age > 1 year were independently associated with increased recurrence risk. These findings suggest that particular attention should be paid when operating on older male patients, as they may be inherently more susceptible to recurrence due to greater abdominal wall tension or higher levels of physical activity. The laparoscopic approach was identified as a protective factor against recurrence in our cohort. However, this observation should be interpreted cautiously because surgical approach selection was not randomized. Differences in case complexity and surgeon preference may have influenced the distribution of surgical approaches and therefore may confound comparisons between techniques. Several previous meta-analyses have similarly suggested that both open and laparoscopic approaches can achieve excellent outcomes when performed by experienced surgeons. Since controversy persists regarding differences in recurrence rates between the open and laparoscopic approaches, additional studies are needed to further clarify this issue (17, 26).

### Limitations

This study has several limitations that should be acknowledged.

First, this was a retrospective, single-center study, which inherently introduces the risk of selection bias. The choice of surgical approach was determined by the operating surgeon based on clinical presentation and predefined operative considerations rather than randomized allocation. Therefore, differences observed between surgical approaches may be influenced by case selection, particularly in more complex or urgent cases such as incarcerated hernia.

Second, the laparoscopic technique used in this study represents a specific approach, and the findings may not be directly generalizable to other laparoscopic techniques. In addition, variations in surgeon experience and technical proficiency across the study period may have influenced operative outcomes.

Third, although the cohort was large, postoperative complications were rare, and detailed analysis of complication timing was limited. Furthermore, long-term follow-up data were not systematically available for all patients, which may limit the assessment of late complications and long-term recurrence.

Fourth, certain clinical variables, such as the size of the hernia sac, degree of ring dilation, or detailed intraoperative technical factors, were not available in the medical records, limiting further in-depth analysis.

Finally, as this study was conducted in a high-volume tertiary pediatric center, the results may not be fully generalizable to institutions with different levels of surgical expertise or resource availability.

## Conclusion

This large single-center cohort study provides valuable insights into the epidemiology and surgical management of pediatric inguinal hernia. Age, sex, and hernia laterality were important determinants of disease presentation and complication risk. The laparoscopic surgery demonstrated favorable perioperative outcomes and offered a clear advantage in detecting contralateral hernias. However, because the choice of surgical approach was not randomized, differences between the open and laparoscopic approaches should be interpreted cautiously. Rather than indicating the universal superiority of one technique, our findings highlight the importance of individualized surgical decision-making. Future prospective studies are warranted to further clarify the comparative effectiveness of different surgical strategies in pediatric inguinal hernia.

## Data Availability

The original contributions presented in the study are included in the article/Supplementary Material, further inquiries can be directed to the corresponding author.
