# Supplementary material for: A 10-year review of pediatric inguinal hernia management at a tertiary center
Source: Front Pediatr. 2026 Apr 20;14:1802302. doi: 10.3389/fped.2026.1802302 (PMC13136119; doi:10.3389/fped.2026.1802302)
Supplement: Supplementary file 1 [file Supplementaryfile1.docx]

Supplementary table 1 Clinical Characteristics of Patients with Inguinal Hernia

| Variables | Total  (n = 9590) |
| --- | --- |
| Sex |  |
| Male | 6928 (72.2) |
| Female | 2662 (27.8) |
| Age^*^ | 2y10m (16d-17y) |
| Type of hernia |  |
| Recurrent hernia | 137 (1.4) |
| non-Recurrent hernia | 9453 (98.6) |
| Metachronous contralateral hernia |  |
| Yes | 99 (1.0) |
| No | 9491 (99.0) |
| Incarcerated hernia |  |
| Yes | 398 (4.2) |
| No | 9192 (95.8) |
| History of abdominal surgery |  |
| Presence | 178 (1.9) |
| Absence | 9412 (98.1) |
| Side of hernia |  |
| Unilateral | 5278 (55.0) |
| Left | 2071 (21.6) |
| Right | 3207 (33.4) |
| Bilateral | 4312 (45.0) |
| Type of surgery |  |
| Open | 649 (6.8) |
| Laparoscopic | 8931 (93.1) |
| Laparoscopic to Open^#^ | 10 (0.1) |
| Operative time (minutes) | 29 (10-188) |
| Synchronous contralateral hernia |  |
| Presence | 3508 (36.6) |
| Absence | 6082 (64.4) |
| Surgical complications |  |
| Presence | 11 (0.1) |
| Absence | 9569 (99.9) |
| Length of postoperative hospital stay (days) | 1 (1-28) |

^*^d: days, m: months, y: years

^#^Conversion from the laparoscopic surgery to the open surgery

Supplementary table 2 Clinical Characteristics of Patients with Recurrent Hernia

| Variables | Recurrent hernia  (n = 137) |
| --- | --- |
| Sex |  |
| Male | 116 (84.7) |
| Female | 21 (15.3) |
| Age^*^ | 2y10m (16d-17y) |
| Recurrence count |  |
| 1 | 127 (92.7) |
| 2 | 10 (7.3) |
| Recurrence interval (days) | 266 (2-3391) |
| Side of hernia before recurrence |  |
| Unilateral | 124 (90.5) |
| Left | 54 (39.4) |
| Right | 70 (51.1) |
| Bilateral | 13 (9.5) |
| Side of hernia after recurrence |  |
| Unilateral | 120 (87.6) |
| Left | 52 (38.0) |
| Right | 68 (49.6) |
| Bilateral | 17 (12.4) |
| Type of surgery before recurrence |  |
| Open | 56 (40.9) |
| Laparoscopic | 81 (59.1) |
| Type of surgery after recurrence |  |
| Open | 24 (17.5) |
| Laparoscopic | 113 (82.5) |
| Operative time (minutes) | 29 (10-188) |
| Surgical complications |  |
| Presence | 1 (0.7) |
| Absence | 136 (99.3) |
| Length of postoperative hospital stay (days) | 1 (1-28) |

^*^d: days, m: months, y: years
